# Green banana pasta diet prevents oxidative damage in liver and kidney and improves biochemical parameters in type 1 diabetic rats

**DOI:** 10.1590/2359-3997000000152

**Published:** 2016-02-11

**Authors:** Aline Rodrigues da Silva, Cláudio Daniel Cerdeira, Anelise Rigoni Brito, Bruno Cesar Correa Salles, Gabriela Franzin Ravazi, Gabriel de Oliveira Isac Moraes, Luciana Rosa Alves Rufino, Rafaela Bergmann Strada de Oliveira, Gérsika Bitencourt Santos

**Affiliations:** 1 Laboratório de Pesquisa em Ciências Biológicas Universidade José do Rosário Vellano Alfenas MG Brasil Laboratório de Pesquisa em Ciências Biológicas, Universidade José do Rosário Vellano (Unifenas), Alfenas, MG, Brasil; 2 Departamento de Bioquímica Instituto de Ciências Biomédicas Universidade Federal de Alfenas Alfenas MG Brasil Departamento de Bioquímica, Instituto de Ciências Biomédicas, Universidade Federal de Alfenas (Unifal), Alfenas, MG, Brasil; 3 Departamento de Análises Clínicas e Toxicológicas Faculdade de Ciências Farmacêuticas Unifal Alfenas MG Brasil Laboratório de Bioquímica Clínica, Departamento de Análises Clínicas e Toxicológicas da Faculdade de Ciências Farmacêuticas, Unifal, Alfenas, MG, Brasil; 4 Laboratório de Biologia e Fisiologia de Micro-organismos Unifenas Alfenas MG Brasil Laboratório de Biologia e Fisiologia de Micro-organismos, Unifenas, Alfenas, MG, Brasil

**Keywords:** Diabetes mellitus, green banana pasta, oxidative damage

## Abstract

**Objective:**

In this study, the effects of a green banana pasta diet on the oxidative damage from type 1 diabetes mellitus (DM) were investigated.

**Materials and methods:**

Formulations containing 25 (F25), 50 (F50), and 75% (F75) of green banana pasta were prepared and included in a 12-week diet of Wistar rats with alloxan-induced type 1 DM. The effects of these formulations in preventing oxidative damage in kidneys and liver homogenates of rats were evaluated using the TBARS assay (lipid peroxidation in liver) and the DNPH assay (protein oxidation in liver and kidneys). Furthermore, the effects of the formulations on the fasting glycemia, fructosamine levels, renal function (creatinine), liver function (enzymes aspartate aminotransferase [AST] and alanine aminotransferase [ALT]), and lipid profile (total cholesterol and fractions) in the serum of rats were evaluated in addition to the evaluation of the centesimal composition and microbiological analysis of the produced green banana pasta.

**Results:**

An F75 diet prevented hyperglycemia in diabetic rats (*p* < 0.05) compared to the diabetic rats fed a standard diet (commercial feed). Notably, the protein oxidation in both the liver and kidneys were prevented in diabetic rats on the F50 or F75 diets compared to the control group, whereas the lipid peroxidation was only prevented in the liver (*p* < 0.05). Moreover, all formulations prevented an increase in the amount of triglycerides in the serum of the rats. The F25 and F50 diet prevented the increase of cholesterol, and the F75-based diet of ALT and fructosamine (*p* < 0.05) supported the anti-hyperglycemic effects and the protection against oxidative damage.

**Conclusion:**

The green banana pasta (F75) diet showed great potential for preventing complications associated with diabetes.

## INTRODUCTION

Diabetes mellitus (DM) is a group of endocrine and metabolic diseases markedly characterized by hyperglycemia. DM is considered a serious public health problem and has frequently been associated with high rates of morbidity and mortality worldwide. The genesis of DM can occur due to defects in insulin secretion by the pancreas, defects in insulin’s target cells (insulin resistance), or a combination of both events (1,2).

Oxidative stress, inflammation, and the formation of advanced glycation end-products (AGEs) have been reported to be long-term complications of DM (3,4). Hyperglycemia and the consequent nonenzymatic glycation of protein, lipids, and nucleic acids generate the AGEs, which are a heterogeneous group of molecules that can cause damage, dysfunction, and organ failure, especially in the eyes, kidneys, liver, nervous system, heart, and blood vessels (3,4).

Hyperglycemia is a stimulus for the increased production of superoxide anions (O_2_^-•^) by endothelial cells in small and large blood vessels and the myocardium. Mitochondrial overproduction of O_2_^- •^ leads to oxidative stress, causing serious damage to cells, organs, and tissues (2). Furthermore, hyperglycemia can result in the priming of neutrophils, generating excessive amounts of O_2_^- •^ through the nicotinamide adenine dinucleotide phosphate (NADPH) oxidase complex during the oxidative burst with the consequent formation of other reactive oxygen (ROS) and nitrogen (RNS) species (5). Lipid peroxidation, protein oxidation, damage to deoxyribonucleic acid (DNA), and the irreversible formation of AGEs caused by ROS are exacerbated due to the decrease in an antioxidant defense with a marked reduction of glutathione (GSH) (2,4).

Among the strategies for the prevention and treatment of DM are the practice of regular physical activities*,* a controlled diet, and pharmacological approaches, such as the use of hypoglycemic agents, insulin therapy, or a combination of both drugs. The positive effects of a healthy diet on the risk of diseases are widely recognized; thus, functional foods (*i.e.,* foods that show a health benefit beyond basic nutrition, providing health promotion or disease prevention) are considered important factors in the prevention and control of DM (6-9).

*Musa x paradisíaca *L. (Musaceae family), commonly known as the banana, originated in Southeast Asia, and currently, over 1,000 varieties of bananas are grown in tropical and subtropical regions around the world (9,10). Bananas are the fourth most important food in trade worldwide (11). Among the varieties of bananas, one of the most common in Brazil is the “Prata” variety, occupying approximately 60% of the cultivated area in Brazil (12,13).

During the process of harvesting and trading bananas in Brazil, approximately 10% of the total production is lost due to the population consuming only ripe fruit and also because the banana is a climacteric fruit. These losses could be reduced by processing the pulp of unripe bananas because an alternative is their transformation into green banana pasta (13-16).

Green banana pasta provides an important alternative for restrictive diets as an important functional food, offering a nutritional increase compared to the ripe fruit that is usually consumed by the population. The primary nutrient of green banana pasta is resistant starch, but potassium, fibers, minerals, vitamins B1 and B6, β-carotene, and vitamin C are present in significant concentrations (13).

The beneficial effects on colorectal cancer, cardiovascular disease, gastrointestinal ulcers, and dyslipidemias have been attributed to the use of green bananas (17). A gluten-free green banana pasta, developed by Zandonadi and cols. (18), has been considered a viable alternative food for patients with celiac disease. In addition, due to its low glycemic index, green banana pasta has better digestion and absorption effects. Thus, the amount of glucose released into the blood occurs gradually, maintaining controlled blood glucose levels without requiring the excessive release of insulin (14,19).

Studies have shown the importance of the banana for DM control (8,9); however, to the best of the authors’ knowledge, there are no reports on the effects of green banana pasta on DM complications. Among the possible metabolites related to DM control, the flavonoids are the most cited, and their antioxidant effects can be implicated on the modulation of oxidative damage in several inflammatory diseases (8,9).

Based on this information, the objective of this study was to evaluate the ability of a dietary approach (different formulations of green banana pasta) on the prevention of oxidative damage from complications of type 1 DM in the liver and kidneys of rats. The effects of these formulations on a possible improvement of biochemical parameters were also investigated by evaluating the fasting glycemia, fructosamine, renal function (creatinine), liver function (AST and ALT), and lipid profile (total cholesterol and fractions) in diabetic rats. In addition, a microbiological quality control and composition analysis of the produced green banana pasta was performed.

## MATERIALS AND METHODS

### Green banana pasta

The production of the green banana pasta was performed in the Dietetics Technical Laboratory of the José do Rosário Vellano University (Unifenas). Twelve banana units (commercial, “Prata” variety) were selected with an average size from 9 to 15 cm and visually classified as “green bananas”. The bananas were cleaned with hypochlorite in a proportion of 200 ppm. Then, they were placed in a pressure cooker and covered with drinking water for 8 minutes after the start of pressure. After that, the bananas were mashed (peel and pulp), and a homogeneous mix was obtained, termed “green banana pasta” in this study. The green banana pasta was stored at a temperature of 10 ºC until the time of the analyses.

### Formulations

The formulations were prepared in accordance with different concentrations of green banana pasta added to the commercial feed for rats, as shown in Chart 1.

**Chart 1:** Prepared formulations containing different concentrations of green banana pasta

Formulation	Commercial feed* (g)	Green banana pasta (g)	Abbreviation
Green banana pasta	0	100	–
Standard	100	–	–
Green banana pasta 25%	75	25	F25
Green banana pasta 50%	50	50	F50
Green banana pasta 75%	25	75	F75

* Composition of commercial feed (commercial ration): Soybean meal, ground whole corn, dextrin, rice husk, wheat bran, rice bran, refined soybean oil, meat and bone meal, fish meal, limestone, sodium chloride (salt common), magnesium oxide, iron sulfate, copper sulfate, manganese monoxide, zinc oxide, calcium iodate, cobalt sulfate, sodium selenite, vitamin A, vitamin D3, vitamin E, vitamin K3, vitamin B1, vitamin B2, niacin, pantothenic acid, vitamin B6, folic acid, biotin, vitamin B12, choline chloride, lysine, methionine, propionic acid. Gene donor species: *Agrobacterium* *tumefaciens* and/or *Arabidopsis thaliana* and/or *Bacillus thuringiensis* and/or *Streptomyces viridochromogenes *and/or *Zea mays.*

### Microbiological analysis

All microbiological analyses were performed according to the methodologies proposed by Silva and cols. (20).

#### Preparation of samples and dilutions

Aliquots of the formulations (25 g) were added to the saline solution (0.85%), forming the dilution 10^-1^ and then the successive dilutions (from 10^-2^ to 10^-5^). With these samples, the microbiological tests listed in Chart 2 were performed, and the results are reported as colony-forming units (CFU)/g samples.

**Chart 2:** Microbiological analysis

Finality	Method/Technique	Agar/broth	Incubation time
Detection of total mesophilic aerobic bacteria	Plate Count /Pour Plate	PCA	28 ºC up to 48 h
Detection of yeasts and molds	Plate Count	Potato Dextrose Agar	25 °C up to 5 days
Detection of Coliforms a 35 ^0^C e 45 ^0^C	MPN	LST broth containing inverted Durham tube	35,5 ºC for 48 h
Detection of *Staphylococcus *Coagulase Positivo	Plate Count	BP Agar/BHI broth	37 ºC for 48 h/37 ºC for 24 h
Detection of *Salmonella *sp.	Plate Count	Hektoen/Agar *Salmonella-Shigella*	37 ºC for 24 h
Detection of *Bacillus cereus*	Plate Count	MYP Agar	30 ºC up to 45 h

LST = Lauryl Sulfate Tryptose; MPN = Most probable number; PCA = Plate Count Ágar; BP = Baird-Parker; BHI = Brain Heart Infusion; MYP = Mannitol Yolk Polymyxin.

## Analysis of the centesimal composition

The centesimal compositions of the green banana pasta and its formulations were evaluated according to AOAC (21). The moisture was determined through the gravimetric method with heat employment based on the weight loss of the material at 105 °C until a stable weight be reached. For the ethereal extract, we used the method of “Soxhlet” (gravimetric) based on the weight loss of the material submitted to extraction with ether or the amount of solubilized material through the solvent. The crude protein was determined by the method of “Kjeldahl” by determining the food nitrogen. The fiber fraction was determined using the gravimetric method after hydrolysis in an acidic medium. The fixed mineral residue (ash) was determined by previous carbonization of the samples followed by incineration in a muffle furnace at 550 °C (21).

## Experimental procedures

### Ethics statement

All animal experiments were conducted in strict accordance with the recommendations of the Guide for the Care and Use of Laboratory Animals (National Institutes of Health [NIH], Washington DC: The National Academy Press, 2011). This study was approved by the institutional ethics committee on the use of animals (CEUA) of the Unifenas (committee opinion No. 23A/2013).

### Animals

In this study, 60 healthy *Rattus norvegicus* (Wistar rats, male, adult, 180-300 g weight, 6-8 weeks old) from Unifenas breeding colonies were used. The animals were housed under standard laboratory conditions (25 ± 1°C) maintained in collective cages containing three individuals each and fed with a specific diet and water *ad libitum* for them to undergo a period of acclimatization (10 days) before being used in the procedures.

### Induction of type 1 diabetes mellitus (DM)

**Figure 1:**
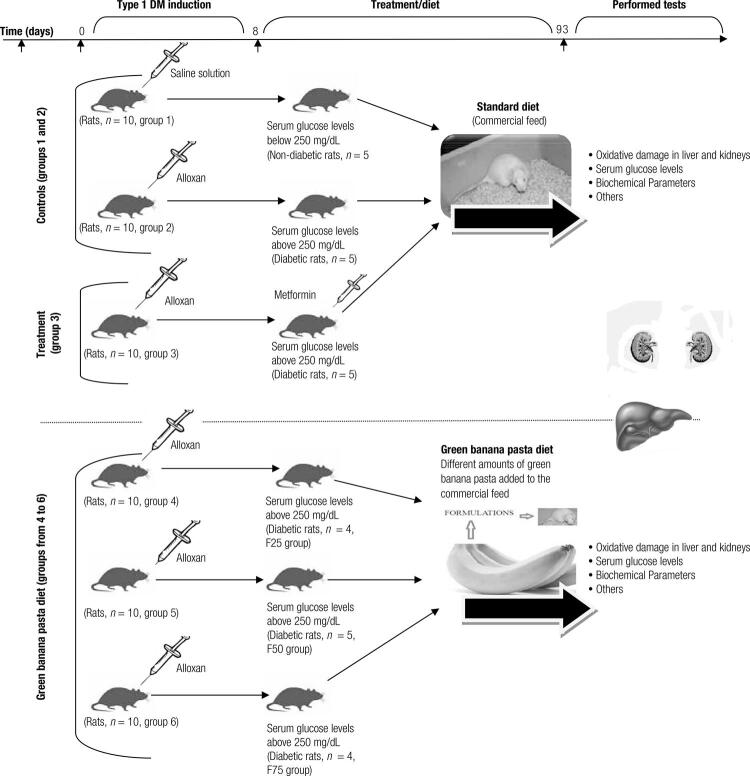
Outline of the experimental design for a 12-week diet of the evaluated experimental groups Subtitle: F25 = Formulation containing 25% of Green banana pasta; F50 = Formulation containing 50% of Green banana pasta; F75 = Formulation containing 75% of Green banana pasta. At first, 60 healthy *Rattus norvegicus* (Wistar rats, male, adult, 180-300 g initial weight, 6-8 weeks old) were divided into six groups, with 10 animals per group. Next, using alloxan (150 mg/kg, intraperitoneally, single dose) type 1 diabetes mellitus (DM) was induced in the groups from 2 to 6 (first week). After that, animals were kept on a standard-style diet or on the formulations-based diets (formulations prepared with different amounts of green banana pasta added to the commercial feed for rats, so-called F25, F50, and F75 diets) for 12 weeks (from day 9 to 93) and then the animals were tested for oxidative damage (in liver and kidneys), serum glucose levels, biochemical parameters, and other tests (after day 93). Treatment with metformin (50 mg/kg/day) for 12 weeks (from day 9 to 93) in the group 3 was performed.

Prior to the induction of type 1 DM through the use of alloxan, the rats were kept in a solid feeding for 12 hours. Then, the animals received a single dose (150 mg/kg, intraperitoneally) of a solution of alloxan monohydrate (Sigma-Aldrich Inc., St. Louis, MO, USA). In the control group of rats without DM, the animals received a single dose of saline solution. One and a half hours later, the supply was reintroduced. Seven days later, weighing and the determination of blood glucose were performed. Rats with fasting blood glucose levels above 250 mg/dL were considered diabetic, as shown in Figure 1 (22).

### Experimental design

After the induction of diabetes, the rats were fed a standard-style diet or the formulations-based diets (formulations prepared with different amounts of green banana pasta added to the commercial feed for rats, F25, F50, or F75) for 12 weeks, and then the animals were tested for oxidative damage and biochemical parameters. The animals were divided into six experimental groups, as shown in Chart 3 and Figure 1. Treatment with metformin (50 mg/kg/day for 12 weeks) was used to confirm the type 1 DM alloxan-induced rat model. The weight and feed intake of the different experimental groups of diabetic rats were evaluated during the 12-week diets.

**Chart 3:** Experimental design of the evaluated experimental groups for a 12-week diet

Group			No. of animals	Diet
Controls (1 and 2)	1	Rats without DM	10	Standard ^1^
	2	Diabetic rats^2^	10	Standard
Treatment (3)	3	Diabetic rats + metformin^3^	10	Standard
Green banana pasta diet (from 4 to 6)	4	Diabetic rats + F25	10	Green banana pasta 25%
	5	Diabetic rats + F50	10	Green banana pasta 50%
	6	Diabetic rats + F75	10	Green banana pasta 75%

^1 ^Commercial feed; ^2 ^DM = type 1 diabetes mellitus; ^3 ^Treatment with 50 mg/kg/day for 12 weeks; F25 = Formulation containing 25% of Green banana pasta; F50 = Formulation containing 50% of Green banana pasta; F75 = Formulation containing 75% of Green banana pasta.

### Biological samples

The animals were anesthetized, and the blood was collected by cardiac puncture to obtain the serum. Then, the animals were euthanized, and the liver and kidneys were removed.

### Serum

The blood samples of the rats were collected in siliconized tubes without additives to obtain the serum. After centrifugation at 1500 g for 10 minutes at room temperature, the serum was separated. The serum was used for the determination of the fasting glycemia, fructosamine, renal function, liver function, and lipid profile.

### Euthanasia and preparation of tissue homogenates

After the maintenance of different diets for 12 weeks, the rats were sacrificed, and the homogenates were prepared from the kidneys and liver as described by Jones and cols. (23). The organs were removed and homogenized (at 4 °C) into 0.1 M phosphate buffered saline (PBS, pH 7.2) in proportions of 5 mL/g organ. The homogenate was centrifuged at 3000 g for 10 min at 4 °C, which was the supernatant subsequently used. The rats were humanely euthanized, and every effort was made to minimize suffering.

### Analysis of fasting glycemia, fructosamine serum levels, lipid profile, renal function, and liver function

Biochemical markers were measured using standard methods. The levels of fasting glucose and total cholesterol (TC) or fractions (Triglycerides [TG] and high-density lipoprotein [HDL]) were determined in the serum through the endpoint colorimetric method. The serum creatinine levels (renal function) were determined by the Jaffe method modified using a kit purchased commercially, and the measurement procedure was calibrated with the reference material SRM 914 of the National Institute of Standards and Technology (NIST), making the results trackable to the definitive method (Isotope Dilution Mass Spectrometry). To evaluate the liver function, the levels of the enzymes aspartate aminotransferase (AST) and alanine aminotransferase (ALT) in the serum were determined by the UV-kinetic method. The serum fructosamine was evaluated by the fixed-time kinetic method using semi-automated equipment (Bio 2000) (24).

### Evaluation of lipid peroxidation

The lipid peroxidation was determined by measuring the peroxidation products that reacted to the thiobarbituric acid (TBA) through the thiobarbituric acid-reactive species (TBARS) assay, as described by Winterbourn and cols. (25). Aliquots (150 µL) of the homogenate from the liver were mixed with 1.22 M phosphoric acid (750 μL), deionized water (1350 μL), and TBA (0.67%, 750 μL) and then incubated. Next, the incubation mixture was boiled in water for 1 hour at 95 °C. After cooling in an ice bath (4 °C), methanol (1800 μL), 1M NaOH (200 μL), and the sample (1000 μL) were added to a cuvette. The concentration of TBARS was estimated from the standard curve of malonic dialdehyde (MDA; 1,1,3,3 tetraetoxipropano). The MDA/TBARS were quantified using a Varian Cary Eclipse spectrofluorimetric detector (λ_excitation_ = 532 nm; λ_emission_ = 563 nm). The results were expressed as µmol MDA/mol of protein. The total protein concentration was determined by the Bradford method (26).

### Determination of protein carbonyls (PCO)

The carbonyls in the oxidized proteins were determined by using the 2,4-dinitrophenylhydrazine (DNPH) spectrophotometric assay*.* This assay is based on the reaction of DNPH with protein carbonyls. Aliquots (500 μL) of the homogenate (from liver or kidneys) plus a 10 μM DNPH solution (500 μL) were incubated for one hour with a dripping trichloroacetic acid (TCA, 20%) solution until complete precipitation. After that, an Ethanol/Ethyl acetate solution (500 μL) was added to the mixture, and it was then centrifuged at 600 g for 10 minutes. More ethanol/ethyl acetate solution (500 μL) was added to the resulting pellet, and a centrifugation was again performed (600 g, 10 minutes). Finally, the pellet was dissolved with 1000 μL of 6 M Guanidine, and the absorbance was measured spectrophotometrically at 370 nm (27). The results were expressed as nmol carbonyl/mg total protein. The total protein concentration was determined by the Bradford method (26).

### Determination of total lipid in the liver

The total lipid in the liver homogenates was determined by using the Bligh Dyer method (21), and the results were reported as the total lipids (%) that represented the total lipid percentage per mL of liver homogenate.

## Data analysis

All graphs were constructed using the BioEstat software (Version 5.0), and the results were expressed as the mean ± standard deviation of at least three experiments. The analysis of variance (ANOVA) followed by Tukey’s test for multiple comparisons of means were performed using the BioEstat software (Version 5.0). The means were considered significantly different with *p* values less than 0.05 (α = 0.05).

## RESULTS

### Microbiological and centesimal composition

The results of the centesimal composition and microbiological analysis are shown in Table 1. The acceptable values in the preparations were up to 1.0 x 10^2^ CFU of the yeasts and molds/g sample (BRASIL, Instrução Normativa nº 4, de 23 de fevereiro de 2007).

**Table 1:** Centesimal composition and microbiological analysis of the formulations and green banana pasta

	Sample/Formulation			
	Green banana pasta	Green banana pasta 25%	Green banana pasta 50%	Green banana pasta 75%
**Centesimal composition**				
Moisture (%)	77	54.7	54.7	62,75
Total solids or dry matter (%)	23	47.45	45.3	37,25
Ash (%)	4.53	6.015	7.065	5,37
Crude fiber (%)	5.52	3.19626	3.55238	3,79629
Lipíds (%)	0.505	0.455	0.73	0,685
Proteíns (%)	2.73	2.55	0.85	1,90
**Microbiological analysis**				
Mesophilic Aerobic	----	Absence	Absence	Absence
Coliforms at 35 ^0^C and 45 ^0^C	----	Absence	Absence	Absence
*Staphylococcus* Coagulase Positive	----	Absence	Absence	Absence
*Salmonella* sp.	----	Absence	Absence	Absence
*Bacillus cereus*	----	Absence	Absence	Absence
Yeasts and molds^1^	----	2 X 10^1^	3,5 X 10^1^	3.31 X 10^6^

^1 ^Colony-forming unit (CFU)/g of sample.

### Effects of green banana pasta on glycemia

**Figure 2:**
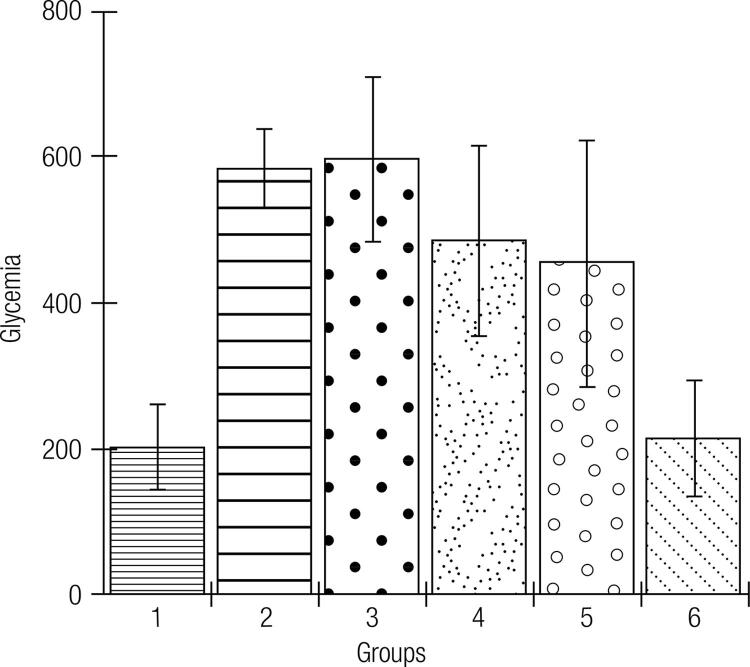
Serum glucose levels (mg/dL) in the different experimental groups.

The serum analyses for the fasting glucose levels of animals on different diets (commercial feed or formulations of green banana pasta) are shown in Figure 2. As observed in Figure 2, an F75 diet shows a significant anti-hyperglycemic effect on type 1 DM, whereas F50 or F25 had no effect on the serum glucose levels. The weights and feed intake for the different experimental groups over the 12-week diets are shown in Table 2.

Subtitle: Experimental groups: 1: Rats without DM + Standard diet; 2: Diabetic rats + Standard diet; 3: Diabetic rats + Standard diet + Metformin; 4: Diabetic rats + F25 diet; 5: Diabetic rats + F50 diet; 6: Diabetic rats + F75 diet; F25 = Formulation containing 25% of Green banana pasta; F50 = Formulation containing 50% of Green banana pasta; F75 = Formulation containing 75% of Green banana pasta; DM = Type 1 diabetes mellitus; ‡: ± standard deviation; Mean values are significantly different when p < 0.05 according to Tukey’s test (α = 0.05); Groups 1, 2, and 3 were maintained with a standard diet (commercial feed) and, in groups 2 and 3, DM was induced by using alloxan (150 mg/kg, intraperitoneally, single dose). Serum glucose levels were of 202.25 ± 58.01 mg/dL in group 1, 584.33 ± 54.55 mg/dL in group 2, and 597.14 ± 113 mg/dL in group 3. Diabetic rats maintained on an F75 diet (group 6) for 12 weeks had lower blood glucose levels (213.5 ± 79.12 mg/dL) compared to animals with DM fed with the standard diet (584.33 ± 54.55 mg/dL) (p < 0.05). Treatment with metformin (50 mg/kg) did not affect serum glucose levels (p > 0.05). An F25-based diet (group 4) or the F50 diet (group 5) did not prevent hyperglycemia in rats with DM induced by alloxan in which the values of serum glucose levels were 484.25 ± 132 (group 4) and 453.8 ± 169 (group 5) mg/dL.

**Table 2:** Weight1 and feed intake2 in the different experimental groups of diabetic rats

	GROUPS				
	Rats without DM + standard diet	Diabetic rats + standard diet	Diabetic rats + F25	Diabetic rats + F50	Diabetic rats + F75
Weight (g)*	----^1^	----	243.325^b^	273.075^a^	263.625^a^
Feed intake (g)**	30.6^a^	39.1^a^	30.25^a^	35.2^a^	39^a^

F25 = Formulation containing 25% of Green banana pasta; F50 = Formulation containing 50% of Green banana pasta; F75 = Formulation containing 75% of Green banana pasta;

* The average weight represents a sample of four rats per experimental group, evaluated on 10 different days;

** Daily feed intake (mean) in the different experimental groups;

^1 ^The weight of a healthy adult rat is approximately 300 g (180-300 g);

^2 ^Feed intake for a healthy adult rat with about 300 g is approximately 30 g. In rats with DM, the polyphagia can increase food consumption. Means followed by same letter in the same row are not significantly different at α = 0.05 (*p* > 0.05).

### Effects of green banana pasta on oxidative damage in the liver and kidneys

**Figure 3:**
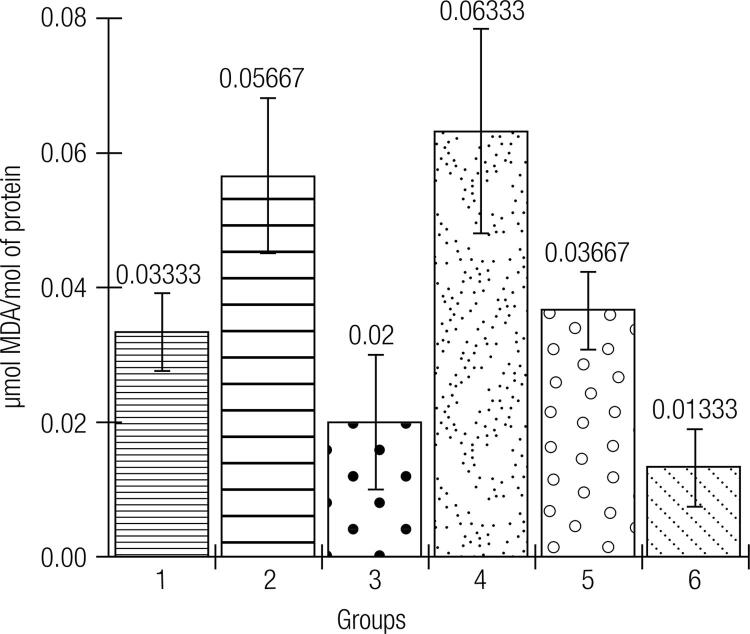
Effects of the formulations of green banana pasta on the lipid peroxidation in the liver of type 1 diabetic rats.

**Figure 4:**
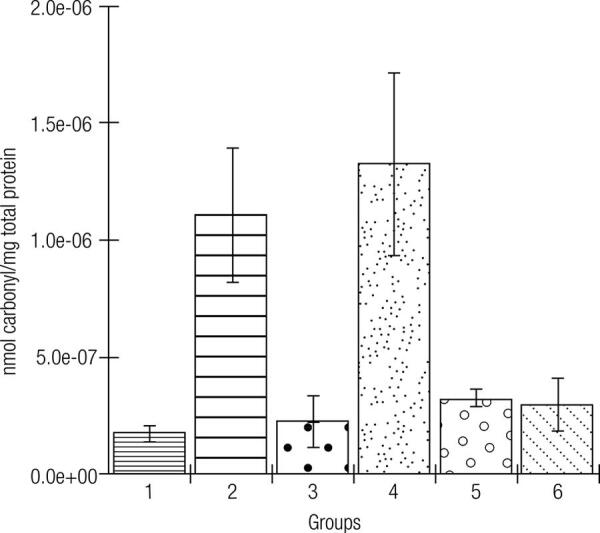
Effects of the formulations of green banana pasta on the protein oxidation in the liver of type 1 diabetic rats.

**Figure 5:**
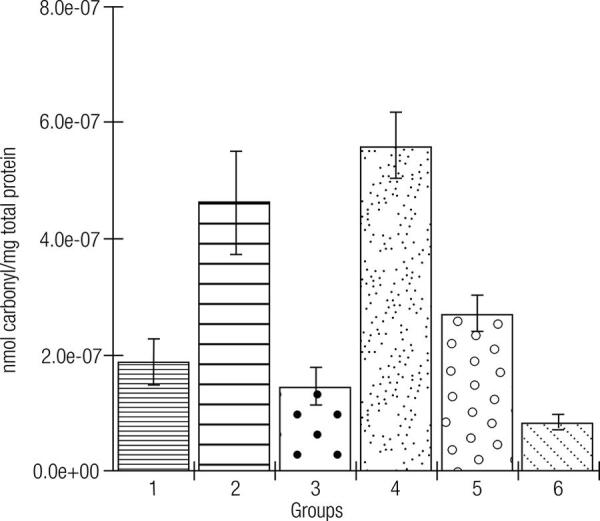
Effects of the formulations of green banana pasta on the protein oxidation in the kidneys of type 1 diabetic rats.

In Figures 3 to 5, the effects of the formulations of green banana pasta on lipid peroxidation (in the liver, Figure 3) and protein oxidation (in the liver and kidneys, Figures 4 and 5, respectively) are shown. As observed, protein oxidation was significantly increased in the liver and kidneys of rats with Type 1 DM that had a standard diet compared to the non-diabetic rats. Notably, the diabetic rats kept on the F50 or F75 diet for 12 weeks showed significantly lower levels of oxidative damage during DM, which is a significant preventative effect of these formulations on lipid peroxidation (in the liver) and protein oxidation (in the liver and kidneys). The animals kept on the standard diet (commercial feed) but treated with metformin had lower levels of lipid peroxidation and protein oxidation compared to the group of untreated animals.

Subtitle: Experimental groups: 1: Rats without DM + Standard diet; 2: Diabetic rats + Standard diet; 3: Diabetic rats + Standard diet + Metformin; 4: Diabetic rats + F25 diet; 5: Diabetic rats + F50 diet; 6: Diabetic rats + F75 diet; F25 = Formulation containing 25% of Green banana pasta; F50 = Formulation containing 50% of Green banana pasta; F75 = Formulation containing 75% of Green banana pasta; DM = Type 1 diabetes mellitus; ‡: ± standard deviation; Mean values are significantly different when p < 0.05 according to Tukey’s test (α = 0.05); Lipid peroxidation was significantly higher in the liver of the diabetic rats kept on a standard diet (commercial feed) than in the liver of rats without DM kept on a standard diet (p < 0.05). Treatment of diabetic rats with metformin (50 mg/kg/day for 12 weeks, group 2) or a 12-week diet with F50 or F75 (groups 5 and 6, respectively) significantly (p < 0.05) prevented lipid peroxidation in the liver of the diabetic rats compared to the animals with DM fed with a standard diet (group 2). F25-based diet (group 5) did not prevent lipid peroxidation in the liver of the diabetic rats compared to the animals with DM fed a standard diet (p > 0.05).

Subtitle: Experimental groups: 1: Rats without DM + Standard diet; 2: Diabetic rats + Standard diet; 3: Diabetic rats + Standard diet + Metformin; 4: Diabetic rats + F25 diet; 5: Diabetic rats + F50 diet; 6: Diabetic rats + F75 diet; F25 = Formulation containing 25% of Green banana pasta; F50 = Formulation containing 50% of Green banana pasta; F75 = Formulation containing 75% of Green banana pasta; DM = Type 1 diabetes mellitus; ‡: ± standard deviation; Mean values are significantly different when p < 0.05 according to Tukey’s test (α = 0.05); Protein oxidation was significantly higher in the liver of the diabetic rats fed a standard diet (commercial feed) than in the liver of rats without DM maintained with the standard diet (p < 0.05). An F50 or F75-based diet of diabetic rats (groups 5 and 6, respectively) for 12 weeks or metformin treatment (50 mg/kg/day for 12 weeks) of the diabetic rats kept on a standard diet (group 3) significantly (p < 0.05) prevented the protein oxidation in the liver compared to the animals with DM maintained with a standard diet (group 2). An F25 diet (group 5) did not prevent protein oxidation in the liver of the diabetic rats compared to the animals with DM fed a standard diet (p > 0.05).

Subtitle: Experimental groups: 1: Rats without DM + Standard diet; 2: Diabetic rats + Standard diet; 3: Diabetic rats + Standard diet + Metformin; 4: Diabetic rats + F25 diet; 5: Diabetic rats + F50 diet; 6: Diabetic rats + F75 diet; F25 = Formulation containing 25% of Green banana pasta; F50 = Formulation containing 50% of Green banana pasta; F75 = Formulation containing 75% of Green banana pasta; DM = Type 1 diabetes mellitus; ‡: ± standard deviation; Mean values are significantly different when p < 0.05 according to Tukey’s test (α = 0.05); Protein oxidation was significantly higher in the kidneys of the diabetic rats fed a standard diet (commercial feed) than in the kidneys of rats without DM fed with the standard diet (p < 0.05). Diabetic rats that had an F50 or F75-based diet (groups 5 and 6, respectively) or metformin treatment (50 mg/kg/day for 12 weeks) of the diabetic rats kept on the standard diet (group 3) had significant preventive effects against the protein oxidation (oxidative damage) in the kidneys (p < 0.05), compared to the animals with DM maintained with a standard diet (group 2). An F25 diet (group 5) did not prevent protein oxidation in the kidneys of the diabetic rats compared to the animals with DM fed a standard diet (p > 0.05).

### Effects of green banana pasta on ALT, AST, total cholesterol and fractions, creatinine, fructosamine, and total lipids (in the liver)

The values of AST, ALT, creatinine, fructosamine, total cholesterol, and fractions (HDL, and triglycerides) in the serum of rats as well as the total lipids in the liver are shown in Table 3. These results demonstrate a significant effect of both the F75 and F50 diet to prevent the increase of ALT in diabetic rats compared to the control group (animals with DM) maintained with the standard diet (*p* < 0.05). An F75 diet was able to prevent the increase of fructosamine in diabetic rats compared to the control group. The animals on an F50-based diet had a significant protective effect against the increase of AST. All formulations significantly prevented the increase in the amount of triglycerides in animals with diabetes (*p *< 0.05), and they had no effect on HDL (*p* = NS). Interestingly, the F50 diet was able to significantly prevent the increase of cholesterol compared to the control group, whereas the F75 diet had no hypocholesterolemic effect. The total lipids in the liver increased in the diabetic rats compared to the non-diabetic rats, whereas for the rats on a green banana pasta diet (F25, F50, and F75 diets) as well as for the rats maintained with the standard diet but treated with metformin, this increase did not occur.

**Table 3:** Serum levels of ALT, AST, creatinine, fructosamine, total cholesterol and fractions (triglycerides and HDL), and total lipids in the liver for the experimental groups

	Rats without DM + standard diet*	Diabetic rats + standard diet	Diabetic rats			
			TREATMENT	DIETS		
			Metformin	Green banana pasta		
				F25	F50	F75
**DOSAGES**						
**Liver function**						
ALT (U/L)	51.7^b^	99^a^	95.4^a^	81^a^	55.4^b^	65.7^b^
AST (U/L)	100.4^b^	141.7^a^	118.7^a,b^	140.7^a^	141^a^	145.4^a^
**Renal function**						
Creatinine (mg/dL)	0.58^a^	0.86^a^	0.88^a^	0.48^a^	0.67^a^	0.57^a^
**Lipid profile**						
Cholesterol (mg/dL)	54^c^	77^a^	74.7^a^	72^a,b^	58^b,c^	76^a^
Triglycerides (mg/dL)	113.7^b^	156.4^a^	151.7^a^	103.5^b^	78^b^	76.7^b^
HDL (mg/dL)	44^a^	49.3^a^	54.5^a^	51^a^	49^a^	43^a^
Total lipids in liver (%)	1.277^b^	1.581^a^	1.092^b^	1.208^b^	1.136^b^	1.109^b^
Marker of hyperglicemy**						
Fructosamine (μmol/L)	113.7^b^	136^a^	126^a^	126^a^	118^a^	110.7^b^

Subtitle: * Standard diet = commercial food; ** Uncontrolled diabetes; Mean values with different letters in the same row are significantly different (p < 0.05; α = 0.05). AST = aspartate aminotransferase; ALT = alanine aminotransferase; HDL = high-density lipoprotein; DM = type 1 diabetes mellitus; F25 = Formulation containing 25% of Green banana pasta; F50 = Formulation containing 50% of Green banana pasta; F75 = Formulation containing 75% of Green banana pasta.

## DISCUSSION

In this study, the effects of a green banana pasta diet on glucose levels during type 1 DM was evaluated in an experimental animal model using Wistar rats with alloxan- induced diabetes. According to Lenzen and Panten, using this model, the animals show symptoms similar to those found in humans (*i.e.,* weight loss, polydipsia, polyphagia, polyuria, glycosuria, ketonuria, hyperglycemia, and ketonemia). Moreover, the complications of DM (*i.e.,* increased oxidative damage and of specific markers) can also be observed. Currently, although the effect of bananas in preventing hyperglycemia has already been reported (8,9), its effects on oxidative damage from type 1 DM remains unknown (28).

In type 1 DM, oxidative stress has been implicated as one of the most frequent chronic complications (29). Protein carbonyls (PCO), considered markers of protein oxidation, as well as the MDA level, a well-known biomarker of lipid oxidative damage, are accordingly considered markers of oxidative stress (25,27). In this study, the significant effect of a green banana pasta diet (F75 and F50) on the prevention of oxidative damage (lipid peroxidation in the liver and protein oxidation in the liver and kidneys) during type 1 DM was shown, although F50 did not show an anti-hyperglycemic effect, indicating a possible pleiotropic effect of these diets.

In accordance with Tiwari and cols., advanced oxidation protein products (AOPPs) are markers of protein oxidation generated during chronic oxidative stress. AOPPs are formed by reactions between oxidants and plasma proteins. The association between the levels of TBARS and AOPPs during DM indicates that proteins are likewise targeted by ROS as the lipids (2). Taking into account the importance of controlling DM complications, preventing oxidative damage is fundamental since the oxidation of proteins affects many physiological functions. Moreover, long-term complications for diabetes patients lead to higher death rates.

The increases in the carbonyl protein levels as well as the AOPPs are associated with structural and conformational changes in proteins, which implies the genesis of certain metabolic diseases and pathological changes, such as diabetic nephropathy. In addition, AOPPs are pro-inflammatory and pro-oxidative compounds that accumulate in patients with DM and can cause endothelial dysfunction and subsequent cardiovascular disease (2). Demonstrating the broad spectrum of damage from oxidative stress during DM, according to Goh and Cooper, the irreversible formation of AGEs can be the result of excessive formation of ROS during hyperglycemia, which is related to the development and progression of DM complications (4).

Diabetes is markedly characterized by hyperglycemia and is associated with dyslipidemia and disturbed liver and kidney functions. To reinforce the evidence of the effects of a green banana pasta diet in preventing the oxidative damage, in this study, the effects of the formulations on the renal function (creatinine) and liver function (ALT and AST) were presented. The evidence of the effects of a green banana pasta diet (F75) in preventing DM complications was strengthened by analyses of the lipid profile (cholesterol, triglycerides, and HDL), fructosamine, ALT, and fat liver content.

An F75 diet was here proved to be able to prevent the increase of fructosamine in animals with DM. Fructosamine is considered a good indicator of metabolic control in DM, usually reflecting the variations of blood glucose in recent weeks. The improvement of this biochemical parameter is remarkable and congruent to the effect of the F75 diet of preventing hyperglycemia, as also demonstrated in this study. Hence, the decrease in the fructosamine levels may be a consequence of the prior anti-hyperglycemic effect of this 12-week diet.

The oxidative damage in the liver was outstanding with a consequent increase of ALT, a more specific marker of liver function. An F75 diet prevented the oxidative damage as well as an increase of ALT serum levels. This formulation showed a preventive effect against the oxidative damage and consequently against the increase of ALT in this organ. The AST levels increased in diabetic rats compared to the rats without DM, but the formulation-based diets had no effect on the prevention of this increase.

Regarding the lipid profile parameters, surprisingly, the F50 diet prevented the increase of cholesterol levels in diabetic rats, whereas the F75 diet had no significant hypocholesterolemic effect. All formulations of green banana pasta significantly prevented an increase in the triglyceride levels in diabetic rats, but none of them significantly altered the HDL levels. Because HDL is considered “good” cholesterol, it appears to be a desirable outcome of these diets. The effects of the formulations on cholesterol and triglycerides can be due to the anti- hyperglycemic effect of the green banana pasta diet (F75 diet), which reinforces its importance in preventing DM complications. During the renal function evaluation, it was observed that the creatinine levels were equal in all experimental groups, although significant protein oxidation and lipid peroxidation in this organ were observed.

Interestingly, metformin showed a good ability in preventing hepatic and renal oxidative damage from type 1 DM, despite it being used for treating type 2 DM. On the other hand, as expected, metformin did not show hypoglycemic effects in animals with type 1 DM. Moreover, it did not prevent the increase of fructosamine and lipid markers, which are derived from hyperglycemia, proving its lack of effect on hyperglycemia and consequent type 1 DM-associated complications. Thus, these findings extend the knowledge on the properties of metformin in reducing oxidative stress, an effect already well-known for the reduction of AOPPs and AGES among other markers of oxidative stress; however, when they are generated during type 2 diabetes (30).

Bananas are a widely consumed food, mainly due to sensory aspects, nutrition, and low cost (13,31). The pulp of green banana has no taste. It contains high starch and is low in sugars and aromatic compounds. The green fruits are rich in flavonoids, also presenting a high content of resistant starch (similar to dietary fiber). The high content of resistant starch hinders the absorption of fats and glucose, so bananas are suggested for patients with DM (19).

In this study, all evaluated formulations of green banana pasta showed considerable amounts of fiber and protein, reinforcing the previous findings on the importance of green bananas (13). Furthermore, the presence of large quantities of fiber is related to important biological effects, such as the positive effects on the gastrointestinal tract and the vascular system as well as hypoglycemic and hypocholesterolemic effects (17). In addition, the evidence linking genus *Musa* to the hypoglycemic effects is based on different studies (8,32,33). Among the metabolites possibly related to this property, the pectin and flavonoids rutin, daidzein, and genistein (known stimulators of insulin secretion) have been frequently reported (34-36). These compounds also have significant antioxidant effects, which could at least partly explain the effect of F50 on the modulation of oxidative damage notwithstanding its inability to have significant anti-hyperglycemic effects on type 1 DM.

Concerning the microbiological analysis, the stable microbiological quality of the formulations was demonstrated. Only the presence of yeasts and molds was evidenced. The increase in the CFU of these microorganisms occurred according to the increase of green banana pasta added to the commercial feed, indicating that their growth may be dependent on humidity.

One of the limitations of this study is the absence of a group of treated diabetic animals with glucose- matched levels to the green banana pasta groups. However, a possible preventive effect of the green banana pasta diets (F75 and F50 diets) directly on the oxidative damage in liver and kidneys is suggested by the high content of antioxidants present in bananas.

Having confirmed and reinforced the link between hyperglycemia and diabetes complications, this study demonstrated that a diet based on formulations containing green banana pasta (F50 or F75 diet) can prevent oxidative damage in the liver and kidneys of rats with type 1 diabetes mellitus (DM). In addition, the F75 diet prevented the increase of ALT, triglycerides (effects also observed for an F50 diet), fructosamine, and glycemia. Therefore, the green banana pasta diet presented promising effects on the prevention of diabetes complications in addition to being considered a nutritious food.
